# Host Species Influence the Gut Microbiota of Endemic Cold-Water Fish in Upper Yangtze River

**DOI:** 10.3389/fmicb.2022.906299

**Published:** 2022-07-18

**Authors:** Liangliang Xu, Peng Xiang, Baowen Zhang, Kun Yang, Fenglin Liu, Zesong Wang, Yanjun Jin, Longjun Deng, Weixiong Gan, Zhaobin Song

**Affiliations:** ^1^Key Laboratory of Bio-Resources and Eco-Environment of Ministry of Education, College of Life Sciences, Sichuan University, Chengdu, China; ^2^Yalong River Hydropower Development Company, Ltd., Chengdu, China; ^3^Observation and Research Station of Sichuan Province of Fish Resources and Environment in Upper Reaches of the Yangtze River, College of Life Sciences, Sichuan University, Chengdu, China; ^4^Institute of Ecology, China West Normal University, Nanchong, China

**Keywords:** gut microbes, host species, core bacteria, cold-water fish, stochastic processes, dietary adaptation

## Abstract

The fish gut microbiome plays an important role in nutrition absorption and energy metabolism. Studying the gut microbes of cold-water fish is important to understand the dietary adaptation strategies in extreme environments. In this study, the gut samples of *Schizothorax wangchiachii* (SW, herbivorous), *Schizothorax kozlovi* (SK, omnivorous), and *Percocypris pingi* (PP, carnivorous) in the upper Yangtze River were collected, and we sequenced 16S rRNA amplicon to study the potential relationship between gut microbes and host species. The results showed that gut microbial composition and diversity were significantly different between the three cold-water fishes. These fishes had different key taxa in their gut microbes, including bacteria involved in the breakdown of food (e.g., *Cetobacterium*, *Aeromonas*, and *Clostridium sensu stricto* 10). The highest alpha diversity indices (e.g., Chao 1 index) were identified in the herbivore (SW), followed by the carnivore (PP), and the lowest in the omnivore (SK). Non-metric multidimensional scaling (NMDS) results revealed that the gut microbial community of these species was different between host species. The neutral community model (NCM) showed that the microbial community structure of SW was shaped by stochastic processes, and the highest species dispersal was found in SW, followed by PP, and the lowest in SK. The results of niche breadth agreed with these findings. Our results demonstrated that host species influenced the gut microbiome composition, diversity, and microbial community assembly processes of the three cold-water fishes. These findings implied that the variation of gut microbiome composition and function plays a key role in digesting and absorbing nutrients from different foods in cold-water fish.

## Introduction

Fish gut microbiota is influenced by trophic level (Liu et al., 2016), season (Neuman et al., 2016; Dulski et al., 2020), host habitat (Dehler et al., 2017; Dulski et al., 2020), and intestinal section (Parata et al., 2020). For example, herbivorous, omnivorous, and carnivorous are the main trophic levels of fish and exhibit differences in gut microbiome composition (Liu et al., 2016; Egerton et al., 2018). Cellulose-degrading bacteria (e.g., *Aeromonas*, *Cetobacterium*, *Leuconostoc*, and *Bacillus*) are dominant in herbivorous fishes (*Megalobrama amblycephala* and *Ctenopharyngodon idellus*), while protease-producing bacteria (e.g., *Cetobacterium* and *Halomonas*) are dominant in carnivorous fishes (*Siniperca chuatsi* and *Culter alburnus*) (Liu et al., 2016). Similarly, this result was found in other fishes (Egerton et al., 2018). The digestive tract of fish is divided into the foregut, midgut, and hindgut. Differences in the composition and diversity of the gut microbiome have been identified between the intestinal sections of few fish species (Tao et al., 2013; Parata et al., 2020; Cheutin et al., 2021). Tao et al. (2013) found that different intestinal sections of *Miichthys miiuy* showed differences in the number of culturable bacterial colonies; the highest number was found in the midgut (27.4%), followed by the foregut (25.2%), and hindgut (22.9%). Furthermore, alpha diversity (Shannon index; 1.5–4.3) of gut microbes in juvenile *Acanthurus triostegus* was higher in the midgut than in the hindgut, while the opposite trend was found in adults (Parata et al., 2020).

Niche-based and neutral theories are two important and complementary mechanisms for understanding the microbial community structure (Sloan et al., 2006; Bahram et al., 2016). Niche-based theories assume that deterministic processes (i.e., deterministic abiotic factors: pH and temperature; biotic factors: competition and predation) shape the microbial community structure (Gipsi et al., 2015; Wei et al., 2016). On the contrary, neutral theories consider that stochastic processes (e.g., birth, death, immigration, and limited dispersal) shape the microbial community structure (Rosindell et al., 2011; Zhou and Ning, 2017). Numerous studies have reported that stochastic processes play an important role in shaping the microbial community structure of the environment (Chen et al., 2019), and in animals (Zhao et al., 2022). However, it is challenging to quantify the relative importance of stochastic processes due to the diversity and complexity of microorganisms. To untangle the relative importance of stochastic processes in microbial community assembly, the neutral community model (NCM) was proposed by Sloan et al. (2006), and it has been widely used for quantifying the importance of stochastic processes (Sloan et al., 2006; Zhao, 2014; Chen et al., 2019).

*Schizothorax wangchiachii*, *S. kozlovi*, and *P. pingi* belong to Cyprinidae and are important commercial cold-water fish distributed in the upper reaches of the Yangtze River and its tributaries (Yue, 2000). Previous studies have shown that the wild population of the three cold-water fishes has decreased due to anthropogenic activities including overexploitation, hydropower development, and water pollution (Jiang et al., 2007; Feng et al., 2011). Thus, multiple studies of population improvement measures such as artificial propagation (He et al., 2014; Zhao, 2014) and stock enhancement (Deng et al., 2016) have been undertaken to protect and improve the resources of these fishes in the wild environment. However, the fry of the three fish species has been mostly fed with mixed feed during artificial breeding (Liu et al., 2015), and this is different for wild populations. For example, *S. wangchiachii* is an herbivorous fish and feeds on periphyton (e.g., diatoms) (Huang, 2018), *S. kozlovi* is an omnivorous fish and mostly feeds on algae (e.g., *Spirogyra* and *Cymbella*) and aquatic insects (e.g., Diptera and Libellulidae) (Zhang and Dai, 2011), and *P. pingi* is a carnivorous fish and feeds on other fishes (e.g., *S. wangchiachii*) (Chen et al., 2015). Consequently, differences in diet composition may influence the survival and growth of the hatchery-reared individuals of these species after stocking for enhancement. Similarly, previous studies showed that the fish gut microbiome played a major role in adapting to dietary changes (Liu et al., 2016). Therefore, the gut microbiome studies of the three cold-water fishes in the wild are helpful to explore the relationship between gut microbes and diet and would provide essential data for stock enhancement programs of the species.

**FIGURE 1:**
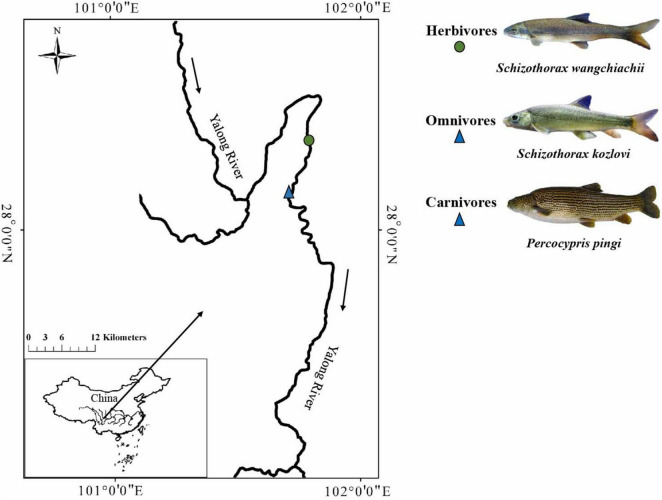
Sampling sites of the three cold-water fishes investigated. The solid circle represents the sampling sites for herbivores, and the solid triangle represents the sampling sites for omnivores and carnivores.

To investigate the potential relationship between the gut microbiome and host species or intestinal sections, we examined the gut samples of three cold-water fishes (*S*. *wangchiachii*, *S*. *kozlovi*, and *P*. *pingi*) from the lower reaches of the Yalong River, a tributary of the upper Yangtze River (Figure 1 and Supplementary Table 1). High-throughput sequencing of the bacterial 16s rRNA gene was used to obtain microbial data. This study was performed to answer the following questions: (1) What is the influence of host species on the gut microbiome of the three cold-water fishes? (2) What is the survival strategy of adapting to different dietary compositions in the three cold-water fishes?

## Materials and Methods

### Sample Collection

Gut samples were collected from individuals of *S. wangchiachii* (SW; foregut: 5, midgut: 5, hindgut: 5), *S. kozlovi* (SK; foregut: 5, midgut: 5, hindgut: 5), and *P. pingi* (PP; foregut: 5, midgut: 5, hindgut: 5) in the lower reaches of the Yalong River, Sichuan Province, China (Figure 1 and Supplementary Table 1). The average water temperature and altitude of the sampling sites (E: 101°86′15, N: 28°27′41 ∼ E: 101°59′91, N: 28°17′23) in the lower reaches of the Yalong River were 11.2^°^C and 1,500 m, respectively. In the sampling water area, drift nets were used to capture fish in April 2021. Each fish was euthanized with MS-222 (0.6–1.0 g/L) after being caught, and its foregut, midgut, and hindgut were cut using sterile scissors. These fishes had a digestive tract that consisted of a foregut: enlarged and with thicker walls; midgut: curved and folded; and hindgut: intestinal diameter gradually narrowed. Based on the digestive tract structure of the three fish species, the intestinal contents from the foregut, midgut, and hindgut were collected. To avoid cross-contamination during sampling, the gut contents were collected from each central part of the foregut, midgut, and hindgut. All gut contents were immediately transferred to 2-ml aseptic centrifuge tubes in the field and stored at –20°C in a portable refrigerator. Finally, all samples of gut contents were stored at –80°C in the laboratory for DNA extraction.

### DNA Extraction and 16S rRNA Sequencing

Gut contents were thawed in ice and then were used to extract DNA by using the QIAamp DNA Stool Mini Kit (Qiagen, Valencia, CA) according to the manufacturer’s instructions. The highly variable V4-V5 region of the 16S rRNA gene was selected and amplified with bacterial-specific universal primers 515F (5′-GTGCCAGCMGCCGCGG-3′) and 907R (5′-CCGTCAATTCMTTTRAGT-3′) (Caporaso et al., 2012). PCR was performed with conditions as follows: initial denaturation at 95°C for 5 min, 35 cycles of 95°C for 30 s, 55°C for 30 s, and 72°C for 45 s; and extension at 72°C for 10 min. The products of PCR amplification were sent to Mingke Biotechnology Co., Ltd. (Hangzhou, China) for high-throughput sequencing on the Illumina HiSeq Platform (Hiseq2500 PE250).

### Sequencing Data Analysis

The QIIME 1.9 software package (Caporaso et al., 2010) was used to process the raw paired-end sequences (2,273,925 reads from 45 samples). The clean sequences (2,134,418) were generated using the trimming analysis and by removing low-quality reads (the base with a mass value below 20 at the tail of read), splicing (the minimum length overlap is 10 bp and the maximum mismatch ratio is 0.2), and quality control (remove chimeras) (Edgar, 2010). After we removed the low-quality reads, the length (bp) distribution of the 45 samples was between 301 and 400 bp (99.96%). Finally, the operational taxonomic units (OTUs) were clustered, with > 97% sequence identity, and each OTU was classified by the annotation against the Silva 132 database (Release132)^1^ (Christian et al., 2013). Sequencing data have been uploaded to the NCBI (accession number PRJNA789150).

### Bioinformatics Analysis

Gut microbial composition (i.e., phylum and genus levels) among different species were compared by using the Mann–Whitney *U*-test and the Kruskal–Wallis *H*-test in Stamp 2.1.3 (Parks et al., 2014). The bar plot was generated by R 2.0 (Kolde, 2015). The linear discriminant analysis (LDA) effect size (LEfSe) method was used to test the differences in the composition of gut microbes of the three fish species (Segata et al., 2011). During the co-occurrence analysis, the relative abundance of bacteria genera was input into Cytoscape 3.4 (Shannon et al., 2003). The plugin CoNet (Faust and Raes, 2016) was used to generate the network plots using these parameters (Spearman index, ρ = 0.5), and the top 60 genera are shown in the plot. The Venn diagram was generated by R software to test the differences in the number of shared and exclusive OTUs among the three fish species.

Alpha diversity was calculated by the Chao 1 index, the Shannon index, a phylogenetic index, and observed OTU numbers. The Mann–Whitney *U*-test and the one−way analysis of variance (ANOVA) were used to analyze the significant differences between intestinal sections (foregut, midgut, and hindgut) of the three cold-water fishes in Stamp 2.1.3 (Parks et al., 2014). The box plot was used to visualize the results. To analyze the differences in the microbial communities between gut samples, we conducted a PERMANOVA (number of permutations: 999) based on three distances (Bray–Curtis distance, unweighted UniFrac distance, and weighted UniFrac distance). The adonis function in the *vegan* package (Dixon, 2003) was used to perform a PERMANOVA on the three distances to obtain the *R*^2^-value (effect size), and the percentage of variation between species. Non-metric multidimensional scaling (NMDS) was used to visualize the beta-diversity results (Anderson, 2010).

The bacterial function of the three cold-water fishes was predicted by PICRUSt (Phylogenetic Investigation of Communities by Reconstruction of Unobserved States) (Langille et al., 2013). The functional profiles of microbial communities were generated by blasting the 16s RNA gene to the Kyoto Encyclopedia of Genes and Genomes (KEGG) database. In the PICRUSt analysis, the significant KEGG pathways (level 2) among species were analyzed *via* the one−way analysis of variance (ANOVA) in Stamp 2.1.3 (Parks et al., 2014). Furthermore, Bonferroni was used to correct the *p*-value (Benjamini and Yekutieli, 2001). The heat map was generated by the heatplus package in R 2.0 (Kolde, 2015) and used to visualize the functional metabolic profiles.

To determine the potential importance of neutral processes in community assembly, the NCM was used to predict the relationship between OTU detection frequency and their relative abundance across the wider metacommunity (Sloan et al., 2006). In the NCM, *Nm* is an estimate of dispersal between communities. The parameter *Nm* determines the correlation between occurrence frequency and regional relative abundance, *N* describes the metacommunity size, and *m* is the immigration rate. The parameter *R*^2^ indicates the overall fit of the neutral model (Sloan et al., 2006). All statistics were analyzed within 95% confidence intervals (CIs) and calculated by bootstrapping with 1,000 replicates. All computations were performed in R 2.0 software.^2^

The niche width approach (Levins, 1968) was used to quantify habitat specialization across the three cold-water fishes in R 2.0 software (see text footnote 2). Greater values of niche breadth indicated that the taxa are widely present and evenly distributed on the large scale, whereas lower values indicated that the taxa occupied fewer habitats and had a disordered distribution. The Kruskal–Wallis *H*-test and the one−way analysis of variance (ANOVA) were used to calculate the significant differences between the niche breadth of the three fish species in Stamp 2.1.3 (Parks et al., 2014).

## Results

### Overview of Gut Samples Data

After data quality processing, we obtained 1,928,859 qualified sequences from 45 samples, an average of 47,232 ± 9,610 sequences per sample. Rarefaction curves showed that the overall quality of sequencing was excellent, and further analysis could be undertaken (Supplementary Figure 1). The gut microbiota of the three cold-water fishes (SW, SK, and PP) were composed of 3,634 OTUs based on 97% sequence similarity. The OTUs were annotated against the Silva 132 database, and 40 phyla, 414 families, and 974 genera were identified (Supplementary Tables 2–4).

### Composition of the Bacterial Community of the Three Cold-Water Fishes

**FIGURE 2:**
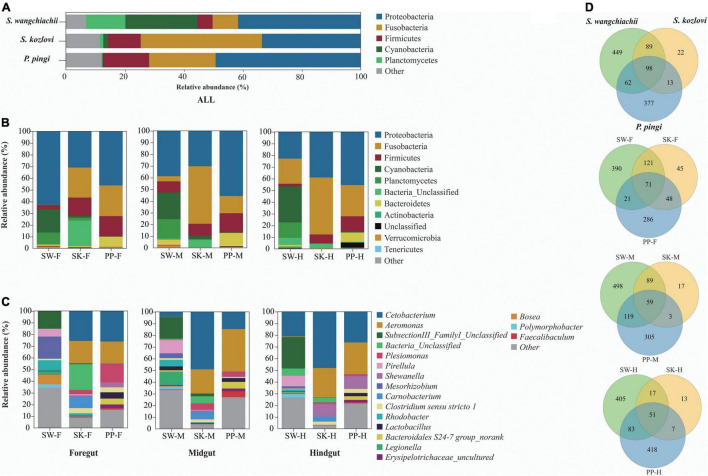
Gut microbiome compositions of the three cold-water fishes. **(A–C)** The bar plot represents the gut microbiome composition of *S. wangchiachii*, *S. kozlovi*, and *P. pingi* among different intestinal sections at phylum and genus levels. **(D)** Venn diagrams based on the OTU level to analyze the differences of gut microbes of SW, SK, and PP. SW, *S. wangchiachii*; SK, *S. kozlovi*; PP, *P. pingi*; F, foregut; M, midgut; H, hindgut.

**FIGURE 3:**
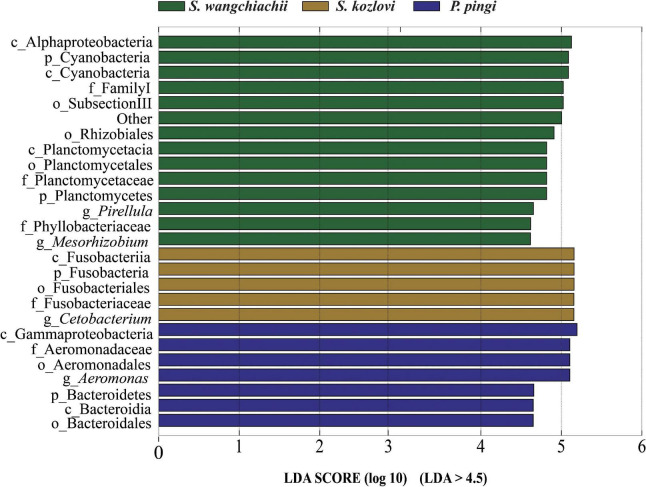
Variation in the composition of the three cold-water fishes. Linear discriminant analysis effect size (LEfSe) analysis of gut microbiota composition of the three fish species (LDA > 4.5).

Significant differences in the gut microbial composition between the three fish species (SW, SK, and PP) were identified (Figures 2, 3 and Table 1). Overall, at the phylum level, the dominant phyla were Proteobacteria (SW: 41.50%; SK: 33.40%; PP: 49.14%) and Fusobacteria (SW: 8.66%; SK: 41.16%; PP: 22.53%) (Figure 2, Supplementary Figure 2, and Supplementary Table 2). We identified significant differences in the gut microbe composition of the three fish species between intestinal sections (foregut, midgut, and hindgut) (Mann–Whitney *U*-test; both, *p* < 0.05) (Figure 2B and Table 1). The relative abundance of Cyanobacteria and Planctomycetes showed significant differences and downward trends between the herbivore (SW), omnivore (SK), and carnivore (PP) (Mann–Whitney *U*-test; both, *p* < 0.05) (Cyanobacteria: SW: 24.39%, SK: 1.61%, PP: 0.17%; Planctomycetes: SW: 13.36%, SK: 1.12%, PP: 0.29%) (Figure 2, Table 1, Supplementary Figure 3, and Supplementary Table 2). In SW vs. SK and SW vs. PP, significant differences in gut microbiome composition were found, while no significant differences were observed in SK vs. PP (Table 1).

**TABLE 1:** Comparison of the relative abundance of gut samples among different host species.

Taxonomy	Different host species		

Phylum	SWF vs. SKF	SWF vs. PPF	SKF vs. PPF
	Foregut		
Cyanobacteria	(0.198 vs. 0.017)**	(0.198 vs. 0.001)**	(0.017 vs. 0.001)^NS^
Fusobacteria	(0.001 vs. 0.256)**	(0.001 vs. 0.262)**	(0.256 vs. 0.262)^NS^
Planctomycetes	(0.102 vs. 0.023)*	(0.102 vs. 0.002)**	(0.023 vs. 0.002)^NS^
Verrucomicrobia	(0.008 vs. 0.001)**	(0.008 vs. 0.001)**	(0.001 vs. 0.001)^NS^
**Genus**			
*Arenimonas*	(0.007 vs. 0.002)^NS^	(0.007 vs. 0.001)**	(0.002 vs. 0.001)^NS^
*Bosea*	(0.084 vs. 0.001)**	(0.084 vs. 0.001)**	(0.001 vs. 0.001)^NS^
*Brevundimonas*	(0.001 vs. 0.000)^NS^	(0.001 vs. 0.004)^NS^	(0.000 vs. 0.004)*
*Clostridium sensu stricto* 10	(0.002 vs. 0.000)*	(0.002 vs. 0.000)*	(0.000 vs. 0.000)^NS^
*Legionella*	(0.026 vs. 0.001)**	(0.026 vs. 0.001)**	(0.001 vs. 0.001)^NS^
*Pirellula*	(0.065 vs. 0.010)*	(0.065 vs. 0.001)**	(0.010 vs. 0.001)^NS^
*Gemmata*	(0.004 vs. 0.001)^NS^	(0.004 vs. 0.001)**	(0.001 vs. 0.001)^NS^
*Mesorhizobium*	(0.187 vs. 0.002)**	(0.187 vs. 0.001)**	(0.002 vs. 0.001)*

**Phylum**	**SWM vs. SKM**	**SWM vs. PPM**	**SKM vs. PPM**
	**Midgut**		

Cyanobacteria	(0.227 vs. 0.028)*	(0.227 vs. 0.002)**	(0.028 vs. 0.002)^NS^
Fusobacteria	(0.044 vs. 0.492)**	(0.044 vs. 0.147)^NS^	(0.492 vs. 0.147)*
Planctomycetes	(0.167 vs. 0.009)**	(0.167 vs. 0.003)**	(0.009 vs. 0.003)^NS^
Verrucomicrobia	(0.017 vs. 0.001)*	(0.017 vs. 0.001)**	(0.001 vs. 0.001)^NS^
**Genus**			
*Arenimonas*	(0.006 vs. 0.001)^NS^	(0.006 vs. 0.001)*	(0.001 vs. 0.001)^NS^
*Bosea*	(0.007 vs. 0.001)**	(0.007 vs. 0.001)*	(0.001 vs. 0.001)^NS^
*Brevundimonas*	(0.001 vs. 0.000)^NS^	(0.001 vs. 0.018)**	(0.000 vs. 0.008)*
*Clostridium sensu stricto* 10	(0.003 vs. 0.001)*	(0.002 vs. 0.000)*	(0.001 vs. 0.000)^NS^
*Legionella*	(0.115 vs. 0.001)**	(0.115 vs. 0.001)**	(0.001 vs. 0.001)^NS^
*Pirellula*	(0.113 vs. 0.001)**	(0.113 vs. 0.001)**	(0.001 vs. 0.001)^NS^
*Gemmata*	(0.008 vs. 0.001)*	(0.008 vs. 0.001)*	(0.001 vs. 0.001)^NS^
*Mesorhizobium*	(0.039 vs. 0.001)**	(0.039 vs. 0.001)**	(0.001 vs. 0.001)^NS^

**Phylum**	**SWH vs. SKH**	**SWH vs. PPH**	**SKH vs. PPH**
	**Hindgut**		

Cyanobacteria	(0.307 vs. 0.003)**	(0.307 vs. 0.002)^NS^	(0.003 vs. 0.002)^NS^
Fusobacteria	(0.215 vs. 0.487)^NS^	(0.215 vs. 0.267)^NS^	(0.487 vs. 0.267)^NS^
Planctomycetes	(0.132 vs. 0.001)*	(0.132 vs. 0.004)*	(0.001 vs. 0.004)^NS^
Verrucomicrobia	(0.001 vs. 0.001)^NS^	(0.001 vs. 0.001)^NS^	(0.001 vs. 0.001)^NS^
**Genus**			
*Arenimonas*	(0.004 vs. 0.001)*	(0.004 vs. 0.001)*	(0.001 vs. 0.001)^NS^
*Bosea*	(0.003 vs. 0.001)*	(0.003 vs. 0.001)*	(0.001 vs. 0.001)^NS^
*Brevundimonas*	(0.002 vs. 0.000)^NS^	(0.002 vs. 0.002)^NS^	(0.000 vs. 0.002)*
*Clostridium sensu stricto* 10	(0.001 vs. 0.000)^NS^	(0.001 vs. 0.000)^NS^	(0.000 vs. 0.000)^NS^
*Legionella*	(0.009 vs. 0.001)*	(0.009 vs. 0.001)**	(0.001 vs. 0.001)^NS^
*Pirellula*	(0.091 vs. 0.001)**	(0.091 vs. 0.001)**	(0.001 vs. 0.001)^NS^
*Gemmata*	(0.006 vs. 0.001)^NS^	(0.006 vs. 0.001)^NS^	(0.001 vs. 0.001)^NS^
*Mesorhizobium*	(0.019 vs. 0.001)*	(0.019 vs. 0.001)**	(0.001 vs. 0.001)^NS^

The Mann–Whitney U-test was used to test the significant differences between the three cold-water fishes. ^NS^P > 0.05; *P < 0.05; **P < 0.001. SW, Schizothorax wangchiachii; SK, Schizothorax kozlovi, PP, Percocypris pingi. F, foregut; M, midgut; H, hindgut.

At the genus level, overall, *Cetobacterium* (SW: 8.53%; SK: 40.95%; PP: 22.38%) and *Aeromonas* (SW: 0.24%; SK: 21.69%; PP: 27.35%) dominated the bacterial composition (Figure 2C and Supplementary Table 4). The relative abundance of *Brevundimonas* showed an upward trend from the omnivore (SK), herbivore (SW), to carnivore (PP) (SK: 0%, SW: 0.04%, PP: 2.6%) (Table 1). Significant differences in the relative abundance of *Bosea*, *Clostridium sensu stricto* 10, and *Pirellula* were identified between the three species (Mann–Whitney *U*-test; both, *p* < 0.05) (Figure 3 and Table 1). For example, *Clostridium sensu stricto* 10 was significantly different between SW-F and SK-F (Mann–Whitney *U*-test; *p* < 0.01) (Table 1). At the OTU level, the highest number of shared OTUs was identified between SW and SK (number: 89), followed by SW and PP (number: 62), and the lowest between PP and SK (number: 13) (Figure 2D). SW had the highest number of unique OTUs (449), followed by PP (377), and the lowest number of unique OTUs in SK (22) (Figure 2D). Among different intestinal sections (foregut, midgut, and hindgut), the highest number of unique OTUs (498) was detected in SW-M, while the lowest number of unique OTUs (13) was recorded in SK-H (Figure 2D).

### Co-occurrence Analysis of the Three Cold-Water Fishes

**FIGURE 4:**
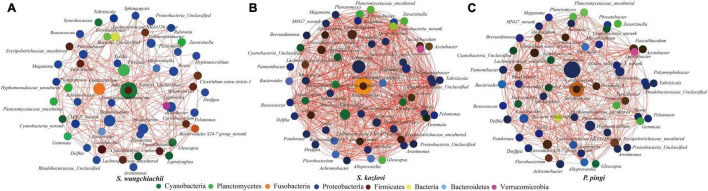
Co-occurrence analysis of the gut microbes (genus level) of the three cold-water fishes (with Spearman index; ρ = 0.5) **(A–C)**. The top 60 genera were used to generate the co-occurrence networks. The node represents the bacteria genus, the size of the node represents the relative abundance, and the color indicates their taxonomic assignment. The red line between the nodes represents a positive correlation, and the blue line indicates a negative correlation.

The co-occurrence analysis showed that the network of gut microbes between the three fish species illustrated distinct co-occurrence patterns (Figure 4). The node in the network diagram mainly belonged to eight phyla: Proteobacteria, Fusobacteria, Firmicutes, Cyanobacteria, Planctomycetes, Bacteria, Bacteroidetes, and Verrucomicrobia (Figure 4). In this study, the bacteria in the center of the co-occurrence were treated as the key taxa (Eiler et al., 2012). *SubsectionIII_FamilyI_Unclassified* (Cyanobacteria) and *Allobaculum* (Firmicutes) were the key taxa in SW, and it tended to be positively correlated with *Pirellula*, *Planctomyces*, *Cyanobacteria_Unclassified*, and *Gloeocapsa* (Figure 4A). The relative abundance of *SubsectionIII_FamilyI_ Unclassified* showed increased trends from foregut (15%) to midgut (18%) to hindgut (27%) (Supplementary Table 4). The same key taxa were found in SK and PP (Figures 4B,C). *Cetobacterium* (Fusobacteria) and *Streptococcus* (Firmicutes) were the key taxa in SK and PP (Figures 4B,C). The higher relative abundance of *Cetobacterium* (the average relative abundance; SK: 41%; PP: 22%) was three and twofold more than *Streptococcus*, respectively (the average relative abundance; SK: 0.09%; PP: 0.10%) (Figures 4B,C and Supplementary Table 4).

### Alpha and Beta Diversity of Gut Microbes of the Three Cold-Water Fishes

**FIGURE 5:**
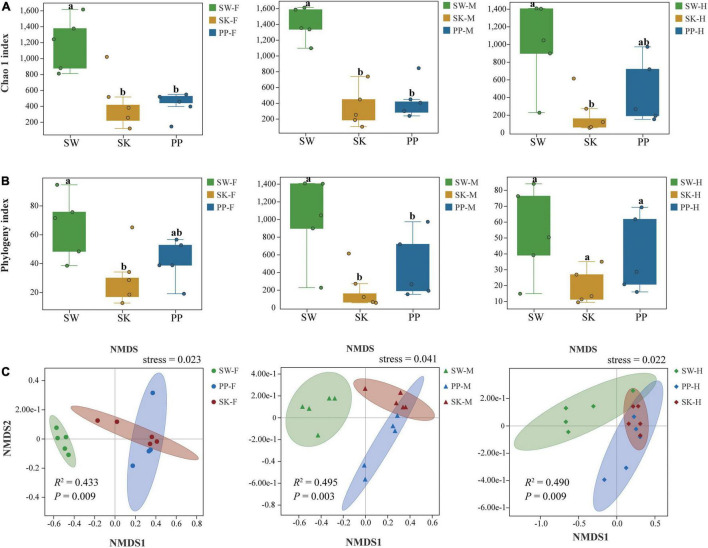
Alpha and beta diversity of gut microbes of the three cold-water fishes. **(A)** The Chao 1 index was calculated for the foregut, midgut, and hindgut of SW, SK, and PP. **(B)** The phylogeny index was calculated for the foregut, midgut, and hindgut of SW, SK, and PP. In the box plot, the top line represents the upper quartile (75th percent), the bottom line represents the lower quartile (25th percent), and the line between the top and bottom lines represents the median. The black points represented the outliers. **(C)** Non-metric multidimensional scaling (NMDS) analysis based on Bray–Curtis distances to explore the dissimilarity in the gut microbes of the three cold-water fishes among different intestinal sections. From left to right: foregut, midgut, and hindgut.

Overall, the Chao 1 and phylogeny indices significantly increased from the omnivorous (SK) to carnivorous (PP) to herbivorous (SW) fish (Kruskal–Wallis *H*-test, *p* < 0.05) (Figures 5A,B and Supplementary Table 5). For example, in the midgut (M), the highest Chao 1 index and phylogenetic index were found in SW (mean ± Sd; Chao 1 index: 1,395 ± 211; phylogenetic index: 77 ± 12), followed by PP (Chao 1 index: 466 ± 237; phylogenetic index: 44 ± 18), and the lowest in SK (Chao 1 index: 343 ± 254; phylogenetic index: 23 ± 12) (Figure 5A and Supplementary Table 5). The two alpha diversity indices showed significant differences between the gut microbes of the three fish species (SW, SK, PP) (Kruskal–Wallis *H*-test, *p* < 0.05) (Figures 5A,B). At the species level, in SW, the highest Chao 1 index was identified in M (midgut) (mean ± Sd; Chao 1 index: 1,395 ± 211), followed by F (foregut) (Chao 1 index: 1,182 ± 339) and H (hindgut) (Chao 1 index: 995 ± 485) (Supplementary Table 5). In SK, F (Chao 1 index: 457 ± 346) had the highest alpha diversity, followed by M (Chao 1 index: 343 ± 254), and the lowest in H (Chao 1 index: 224 ± 237) (Supplementary Table 5). In PP, the highest Chao 1 index was identified in H (Chao 1 index: 459 ± 365), followed by M (Chao 1 index: 446 ± 237) and F (Chao 1 index: 411 ± 160) (Supplementary Table 5).

The gut microbial composition of the three cold-water fishes showed significant differences between different species (Figure 5C and Supplementary Figure 4 and Table 2) (PERMANOVA; both, *p* < 0.05). Among F, M, and H, the NMDS plots showed that the gut microbial communities of SW, SK, and PP were different and could be easily distinguished (Figure 5C). The PERMANOVA test on the three distances had the same result (PERMANOVA; both, *p* < 0.05) (Table 2). Furthermore, we found that the microbial community composition of SK was more similar to PP than to SW (Figure 5C).

**TABLE 2:** Results of the PERMANOVA for the gut samples of the three cold-water fishes.

PERMANOVA						
Type	Sample	Distance	*df*	*F*	*R* ^2^	Bonferroni-corrected *p*-value
Different host species	SW-F	Bray_curtis	2	4.5906	0.43346	0.009
	SK-F	Unweighted_UniFrac	2	2.363	0.28256	0.024
	PP-F	Weighted_UniFrac	2	5.3353	0.47068	0.015
	SW-M	Bray_curtis	2	5.8885	0.49531	0.003
	SK-M	Unweighted_UniFrac	2	3.6984	0.38134	0.003
	PP-M	Weighted_UniFrac	2	5.7687	0.49017	0.003
	SW-H	Bray_curtis	2	5.7729	0.49036	0.009
	SK-H	Unweighted_UniFrac	2	3.1845	0.34673	0.003
	PP-H	Weighted_UniFrac	2	5.7268	0.48835	0.003

*The Kruskal–Wallis H-test was used to analyze the significant differences among different species, and the Bonferroni was conducted to correct the p-value. SW, Schizothorax wangchiachii; SK, Schizothorax kozlovi, PP, Percocypris pingi. F, foregut; M, midgut; H, hindgut.*

### Potential Functional Groups

**FIGURE 6:**
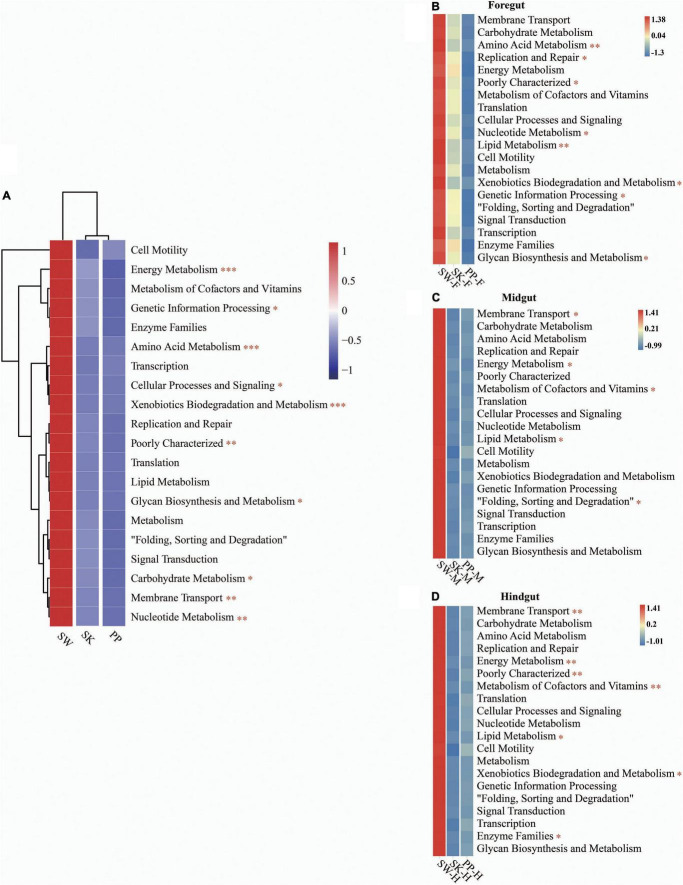
Metabolic functional profiles of the gut microbiome of the three cold-water fishes (KEGG level 2). **(A–D)** Metabolic functional profiles of the gut microbiome of all species, *S. wangchiachii*, *S. kozlovi*, and *P. pingi* among different intestinal sections. F, foregut; M, midgut; H, hindgut. The one-way analysis of variance (ANOVA) was used to identify significant differences between different samples. *p < 0.05; ^**^p < 0.01; ^***^p < 0.001.

Overall, the KEGG pathway analysis (level 2) showed significant differences in the abundance of some diet-related functional categories between the herbivore (SW), omnivore (SK), and carnivore (PP) (one-way ANOVA, both *p* < 0.05) (Figure 6 and Supplementary Figure 5), for example, metabolism of cofactors and vitamins, amino acid metabolism, membrane transport, and lipid metabolism (Figure 6 and Supplementary Figure 5). Overall, a higher abundance of functional categories was found in SW than in SK and PP (Figures 6B–D). Furthermore, significant differences in the gut microbiota of the three fish species between intestinal sections (foregut, midgut, and hindgut) represented differences in KEGG pathways (level 2) (one-way ANOVA, both *p* < 0.05) (Figure 6). A higher abundance of metabolism-related functional categories was identified in the midgut (e.g., SW-M and PP-M) than in the foregut and hindgut (Figure 6).

### Microbial Community Assembly Processes of the Three Cold-Water Fishes

**FIGURE 7:**
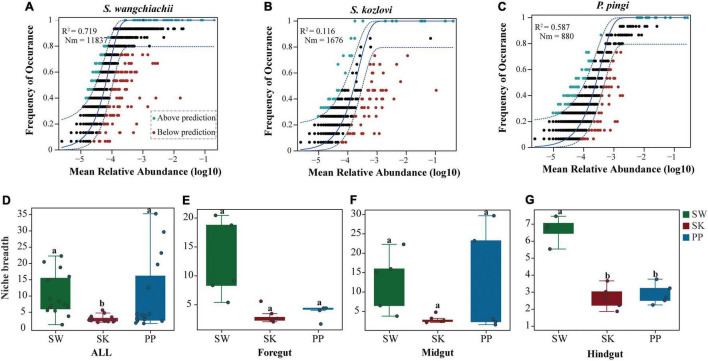
Community assembly process measurements using the niche width and neutral community model (NCM) of the three cold-water fishes. **(A–C)** Predicted occurrence frequencies of *S. wangchiachii*, *S. kozlovi*, and *P. pingi* gut microbes, respectively. The solid blue line represents the best fit to the NCM, and the dashed blue line indicates 95% confidence intervals around the model prediction. The green point indicates OTUs that occur more frequently than predicted by the NCM, and the red point represents OTUs that occur less frequently than predicted by the model. *R*^2^ represents the fit to this model, and *Nm* represents the metacommunity size times immigration. **(D–G)** The relative abundance of the niche width of the three cold-water fishes. SW, *S. wangchiachii*; SK, *S. kozlovi*; PP, *P. ping.*

The NCM successfully estimated the relationship between the occurrence frequency of OTUs and their relative abundance variations (Figures 7A–C), with 72, 12, and 56% of explained community variance for SW (*S. wangchiachii*), SK (*S. kozlovi*), and PP (*P. pingi*), respectively. Furthermore, the value of *R*^2^ was higher in SW (*R*^2^ = 0.719) than in PP (*R*^2^ = 0.587) (Figures 7A–C). These results indicated that stochastic processes play a key role in shaping the microbial community assembly in SW. The highest *Nm*-value was found in SW (*Nm* = 11,837), followed by SK (*Nm* = 1,676), and the lowest in PP (*Nm* = 880) (Figures 7A–C). This finding implied that species dispersal of gut microbes was higher in SW than in SK, or PP.

The niche breadth was used to estimate the community-level habitat of the three cold-water fishes (Figures 7D–G). Overall, the highest value of niche breadth was identified in SW (mean ± Sd: 10 ± 7), followed by PP (9 ± 11), and the lowest in SK (3 ± 1) (Figures 7D–G). Moreover, a significant difference in the niche breadth value was found between the three species (Kruskal–Wallis *H*-test, *p* < 0.05) (Figures 7D–G).

## Discussion

### Composition Differences of Gut Microbes of the Three Cold-Water Fishes

Fish gut microbiota plays an important role in nutrition digestion and absorption (Wu et al., 2015; Liu et al., 2016; Li et al., 2017). Previous studies have reported that host feeding on more protein may result in a greater Firmicutes-to-Bacteroidetes ratio of the gut microbiome (De Filippo et al., 2010; Gong et al., 2021). At the phylum level, we found that the Firmicutes-to-Bacteroidetes ratio exhibited an upward trend from SW to SK and PP (Supplementary Table 2). The ratio of protein in food also showed an upward trend from SW to SK to PP (Supplementary Table 1). Therefore, these results implied that the higher Firmicutes-to-Bacteroidetes ratio may help SK and PP to obtain more energy from a protein-rich diet (De Filippo et al., 2010; Gong et al., 2021). Furthermore, the relative abundance of Cyanobacteria in gut microbes significantly decreased from SW to SK and PP (Supplementary Table 2). A previous study showed that Cyanobacteria is an important food source for fish (Currin et al., 2011). Thus, the difference in the relative abundance of Cyanobacteria in the fish gut microbiome is related to the variation in food composition. This result was consistent with our observations. The ratio of algae in food composition showed a downward trend from SW (herbivore) to SK (omnivore) and then to PP (carnivore) (Zhang and Dai, 2011; Chen, 2013; Huang, 2018). Variation in gut microbial composition between species may contribute to digesting food and absorbing nutrients from different dietary compositions (Parata et al., 2020).

Differences in the composition of gut microbes were identified between the three fish species at the genus level (Figure 3 and Table 1). *SubsectionIII_FamilyI_Unclassified* (Cyanobacteria) was the core bacteria in SW (herbivore) (Figure 4A). Cyanobacteria is one of the main food sources for SW (Huang, 2018). Hence, the core genera *SubsectionIII_FamilyI_Unclassified* may play a key role in helping SW to digest and absorb nutrients from Cyanobacteria. Furthermore, some cellulose-degrading bacteria were found in the gut microbes of SW (Supplementary Table 4). These findings were in accordance with those of previous studies (Li et al., 2015, 2017; Liu et al., 2016). *Clostridium sensu stricto* 10 (*C. sensu stricto* 10), and *Clostridium sensu stricto* 13 (*C. sensu stricto* 13) found in herbivores (e.g., grass carp) had the ability to digest xylan, hemicellulose, and cellulose (Uffen, 1997; Uz and Ogram, 2006). Therefore, these bacteria may play an important role in nutrient absorption from periphyton (e.g., diatoms and Cyanobacteria) in SW (Supplementary Table 1). Several protease-producing bacteria (e.g., *Cetobacterium*, *Aeromonas*, and *C. sensu stricto* 1) were observed in SK (omnivore) and PP (carnivore) (Supplementary Table 4). Moreover, *Cetobacterium* and *Aeromonas* were the core bacteria in the gut microbes of SK and PP, respectively (Figures 4B,C). A previous study showed that *Cetobacterium* was the dominant genera in carnivorous fish, with the ability to ferment carbohydrates and peptides (Finegold et al., 2003). *Aeromonas* plays a vital role in nutrient absorption in fish (Namba et al., 2007; Nayak, 2010). The *C. sensu stricto* 1 found in carnivorous and omnivorous fish had the ability to break down proteins (Schwab et al., 2011). Therefore, these protease-producing bacteria (e.g., *Cetobacterium*, *Aeromonas*, and *C. sensu stricto* 1) may help SK and PP to utilize nutrition and harvest energy from protein-rich foods (e.g., aquatic insects and fish) (Supplementary Table 1).

### Variation of Gut Microbiota Diversity Within the Three Cold-Water Fish Species

Host species can influence the alpha and beta diversity of fish gut microbiomes (Liu et al., 2016; Li et al., 2017). These findings agreed with our results, where we found significant differences in alpha and beta diversities of gut microbes between species (Figure 5). Furthermore, the highest alpha diversity indices were found in SW, followed by PP, and the lowest in SK (Figures 5A,B and Supplementary Table 5). Li et al. (2017) also found higher alpha diversity in the herbivore (*M. amblycephala* grass carp) than in the omnivore (*C. carpio* crucian carp) (Li et al., 2017). The higher alpha diversity in SW is likely due to its herbivorous diet, where it feeds on periphyton (e.g., diatoms and Cyanobacteria) (Huang, 2018). The SW needs to swim and forage extensively to acquire more food to meet its growth and reproduction requirements. The gut microbes of SW may acquire more microbes from the aquatic environment than other species and increase the alpha diversity.

Differences in diversity (alpha and beta diversity) of other fish gut microbiomes have been found between intestinal sections (Ye et al., 2014; Parata et al., 2020; Cheutin et al., 2021). The midgut and hindgut microbiomes in surgeonfish (*Acanthurus triostegus*) represented significant differences in the Shannon index and multi-dimensional scaling (MDS) (Parata et al., 2020). In this study, the midgut exhibited the highest diversity indices, followed by the foregut and hindgut Supplementary Table 5). Our results were inconsistent with those of previous studies (Ye et al., 2014; Yang et al., 2019). The results in our study may be due to a combination of factors. The habitat of the three cold-water fishes surveyed was in lower water temperatures and higher altitudes than the warm-water fishes in Yang et al. (2019). Furthermore, the alpha diversity of fish gut microbiomes between intestinal sections may be affected by variables such as pH (in intestinal lumen), food composition, and oxygen concentration (Ye et al., 2014; Yang et al., 2019). Thus, the cold, high-altitude habitat of these fishes may explain the differences found in other studies.

### Compartmentalization of the Functional Differences Between the Three Cold-Water Fishes

The gut microbial function is of great significance in understanding the mechanism of the host adapting to different diets (Gong et al., 2021). The KEGG pathways (level 2) were enriched in diet-related functional categories, such as carbohydrate metabolism, amino acid metabolism, and lipid metabolism that showed differences between species (Figure 6 and Supplementary Figure 5). This result was in agreement with a previous study by Liu et al. (2016) that found that the abundance of carbohydrate metabolism and lipid metabolism showed differences in herbivorous, omnivorous, and carnivorous fishes.

The food compositions were different between the herbivore (SW), omnivore (SK), and carnivore (PP) (Chen, 2013; Zhao, 2014; Huang, 2018; Supplementary Table 1). The periphytic algae were the dominant food of SW and are rich in fatty acids (De Castro Araújoand Garcia, 2005; Huang, 2018). The highest abundance of lipid metabolism was found in SW, followed by PP, and the lowest in SK (Figure 6 and Supplementary Figure 5). Therefore, differences in the relative abundance of lipid metabolism of gut microbes that were identified between species were to adapt to different fatty acid contents of food. The highest abundance was identified in the KEGG pathway of membrane transport in SW. A previous study showed that this pathway may help hosts increase the efficiency of nutrient absorption and adapt to low-temperature environments (Xia et al., 2021). SW (herbivore) is distributed in the upper reaches of the Yangtze River and its tributaries (low water temperature) and feeds on periphytic algae (e.g., diatoms and Cyanobacteria) (Yue, 2000; Huang, 2018). Therefore, the pathway of membrane transport enriched in the gut microbes of SW may play a key role in absorbing nutrients from periphyton and adapting to a low-water temperature environment. In the fish gut microbiome, different intestinal sections (foregut, midgut, and hindgut) play different roles in digesting and absorbing nutrients (Li et al., 2018). It is known that the midgut is the major site of digestion and absorption of nutrients with a higher abundance of diet-related gut microbiome functional categories than other intestinal sections (Chew et al., 2018). In the present study, the abundance of the metabolism pathway (e.g., carbohydrate metabolism, amino acid metabolism, and lipid metabolism) was higher in the midgut than in the foregut and hindgut in the three cold-water fish (Figure 6 and Supplementary Figure 5), which was in accordance with the results of a previous study (Chew et al., 2018). Thus, the gut microbiome of the midgut enriched in diet-related functional categories may play an important role in digesting and absorbing nutrients from the different diets in the three cold-water fishes.

### Variation of Gut Microbiota Assembly of the Three Cold-Water Fishes

Studying gut microbiota assembly of animals is crucial to understanding the contribution of ecological processes to the structure of microbial communities in microbial ecology (Sloan et al., 2006; Yan et al., 2016). The NCM and niche breadth are valid approaches for exploring gut microbiota assembly and have been successfully applied to many studies (Yan et al., 2016; Chen et al., 2019). In this study, from SK to PP and then to SW, the contribution of stochastic processes showed an increased trend in shaping the bacterial community structure (Figures 7A–C). This result was consistent with that of Yan et al. (2016) which was focused on the bacterial community assembly processes of herbivorous (*Ctenopharyngodon idellus*) and carnivorous (*Siniperca chuatsi*, *Silurus meridionalis*) species in China. Their results showed that the bacterial community structure of these fish species was mainly driven by stochastic processes (i.e., drift process) (the relative abundance of drift process: *Ctenopharyngo_ don idellus*: 68%; *Siniperca chuatsi*: 66%; *Silurus meridionalis*: 67%) (Yan et al., 2016).

Moreover, our findings showed that the highest species dispersal was found in SW, followed by PP, and the lowest in SK (Figures 7A–C). Similarly, the results of niche breadth agreed with these findings (Figures 7D–G). This phenomenon may be explained by the different feeding behaviors of the three fish species. SW is herbivorous and feeds on low-nutrient algae (e.g., diatoms) (Huang, 2018) and forages extensively across its habitat seeking its food. However, PP and SK feed mainly on fish and aquatic insects, respectively (Zhang and Dai, 2011; Chen, 2013). Thus, compared to PP and SK, SW requires a larger feeding area to obtain food for growth and reproduction. Overall, these findings illustrated that host species influenced the microbial community assembly processes in the three cold-water fishes.

## Conclusion

The present study demonstrated that host species influenced the gut microbial composition, diversity, function, and assembly processes of the three cold-water fishes. The gut microbiome of these species had different key bacteria and showed significant differences in several bacteria that were involved in nutrient absorption and diet-related functional categories. The highest alpha diversity indices were identified in the herbivore (SW), followed by the carnivore (PP), and then the omnivore (SK). The gut microbial community showed significant differences between different species. The highest species dispersal was found in SW, followed by PP, and the lowest in SK. Overall, the results implied that increasing the relative abundance of food digesting bacteria and changing the abundance of diet-related pathways may be very important for the three cold-water fishes to digest and uptake nutrients from different foods.

## Data Availability Statement

The data presented in this study are deposited in the NCBI repository, accession number PRJNA789150.

## Ethics Statement

The animal study was reviewed and approved by the Sichuan Provincial Department of Agriculture and Rural Affairs; Approval Letter of Sichuan Agriculture (2021).

## Author Contributions

ZS conceived the project. LX performed the experiments. LX, PX, BZ, KY, FL, ZW, YJ, LD, and WG collected the sample. LX analyzed the data. LX and ZS wrote the manuscript. All authors gave final approval for the publication.

## Conflict of Interest

LD and WG were employed by the company Yalong River Hydropower Development Company, Ltd. The remaining authors declare that the research was conducted in the absence of any commercial or financial relationships that could be construed as a potential conflict of interest.

## Publisher’s Note

All claims expressed in this article are solely those of the authors and do not necessarily represent those of their affiliated organizations, or those of the publisher, the editors and the reviewers. Any product that may be evaluated in this article, or claim that may be made by its manufacturer, is not guaranteed or endorsed by the publisher.

## Footnotes

1
http://www.arb-silva.de

2
http://www.R-project.org
