# Supplementary material for: Host Species Influence the Gut Microbiota of Endemic Cold-Water Fish in Upper Yangtze River
Source: Front Microbiol. 2022 Jul 18;13:906299. doi: 10.3389/fmicb.2022.906299 (PMC9339683; doi:10.3389/fmicb.2022.906299)
Supplement: Supplementary file 1 [file Data_Sheet_1.PDF]

Supplementary material

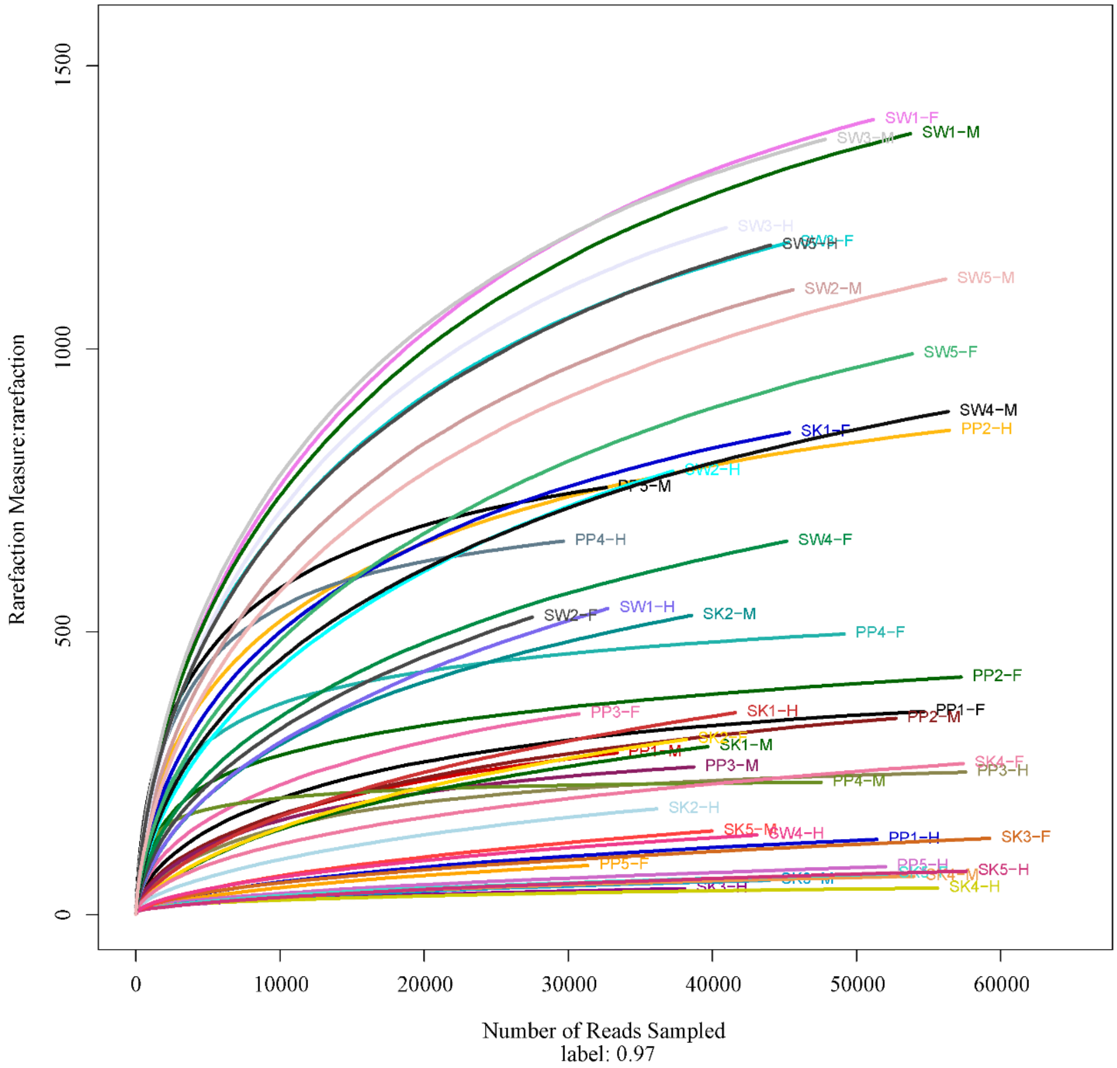

Fig. S1 The rarefaction curves of the 45 samples. SW, *Schizothorax wangchiachii* samples; PP, *Percocypris pingi* samples; SK, *Schizothorax kozlovi* samples; F, foregut; M, midgut; H, hindgut.

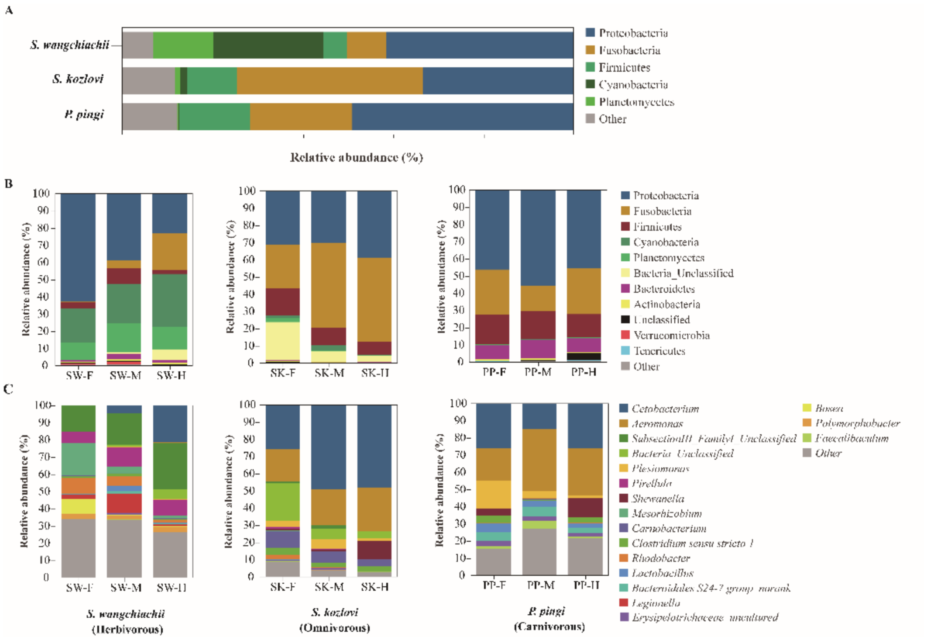

Fig. S2 The gut microbiome composition of three cold-water fishes at the phylum and genus level. SW, *Schizothorax wangchiachii* samples; PP, *Percocypris pingi* samples; SK, *Schizothorax kozlovi* samples; F, foregut; M, midgut; H, hindgut.

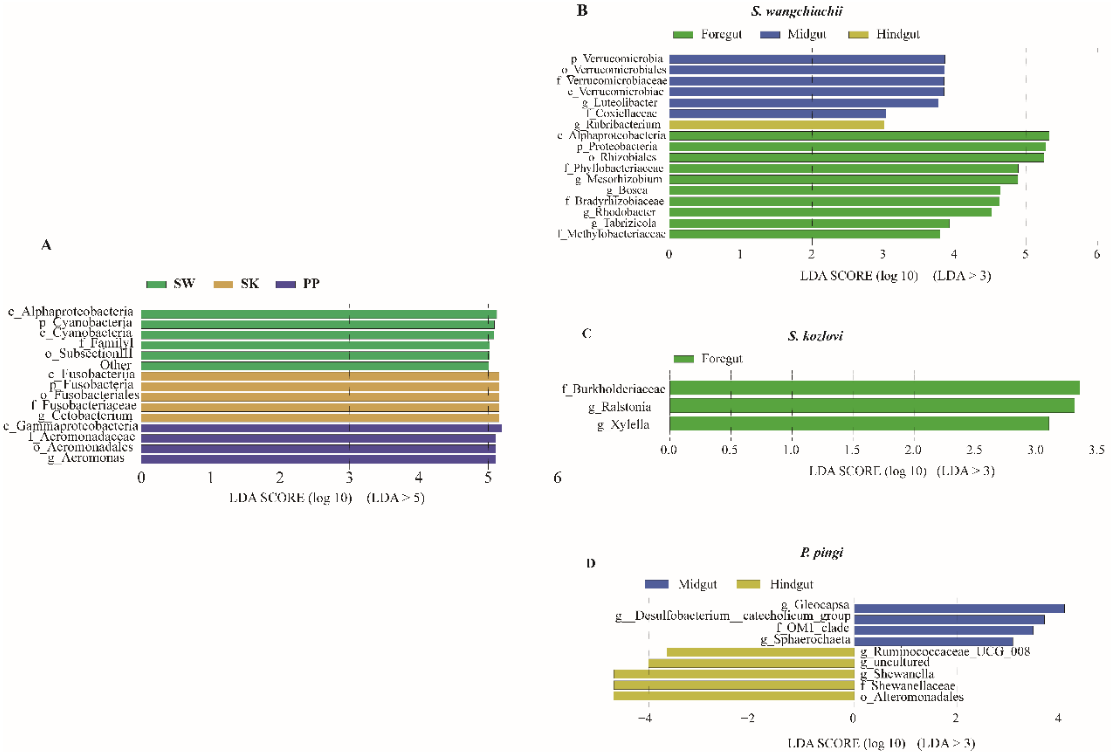

Fig. S3 Variation in the composition of the three cold-water fishes. A, B, C, D: Linear discriminant analysis effect size (LEfSe) analysis of gut microbiota composition of the three fish species

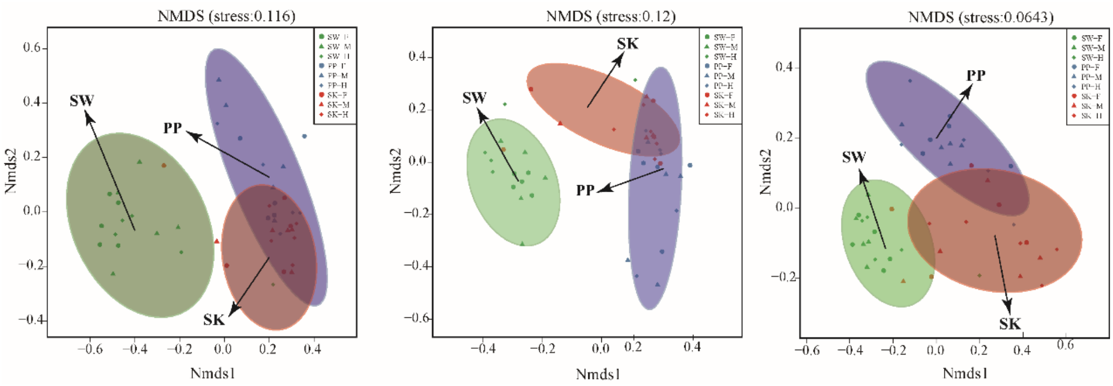

Fig. S4 Nonmetric multidimensional scaling (NMDS) analysis using three distances explored the dissimilarity in the gut microbial of the three cold-water fishes. From left to right**:** bray–Curtis distance, unweighted UniFrac distance, and weighted UniFrac distance. F, foregut; M, midgut; H, hindgut. SW, *Schizothorax wangchiachii*; PP, *Percocypris pingi*; SK, *Schizothorax kozlovi*.

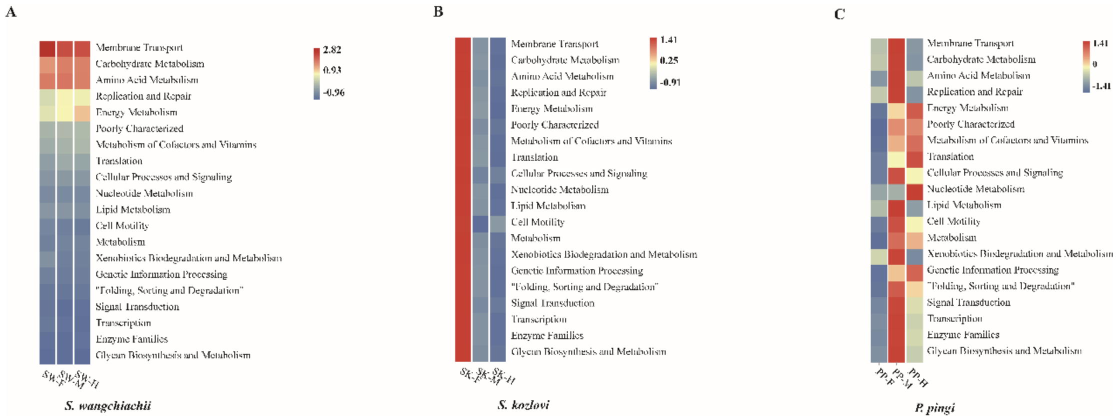

Fig. S5 Metabolic functional profiles of gut microbiome of the three cold-water fishes. (KEGG Level 2). A, B, C: The Metabolic functional profiles of gut microbiome of *S. wangchiachii*, *S. kozlovi*, and *P. pingi* among different intestinal sections. F: foregut; M: midgut; H: hindgut.

Table. S1 The information of the samples obtained from three cold-water fishes from the lower Yalong river.

Species	Age	Weight (g)	Food source	Overall length (mm)	Body length (mm)	Water temperature (℃)	Location	
S.Wangchiachii (SW)	Adult	969	Periphyton	445	366	10.7	101.861489	28.274057
		632		399	332			
		975		418	336			
		802		411	330			
		266		302	242			
S.kozlovi (SK)	Adult	682	Algae and aquat icinsect	415	345	11.7	101.599075	28.172294
		760		410	358			
		673		413	343			
		701		407	352			
		128		235	198			
P. pingi (PP)	Adult	568	Fish	376	335			
		498		381	343			
		523		367	310			
		400		340	286			
		293		387	255			

Table. S2 Gut microbiome composition of the three cold-water fishes among different species and intestinal sections at phylum level (top 10).

Phylum level	*S.Wangchiachii*			*P. pingi*			*S.kozlovi*		
	**Foregut**	**Midgut**	**Hindgut**	**Foregut**	**Midgut**	**Hindgut**	**Foregut**	**Midgut**	**Hindgut**
Proteobacteria	0.629	0.388	0.229	0.462	0.557	0.455	0.309	0.303	0.390
Fusobacteria	0.001	0.044	0.215	0.262	0.147	0.267	0.256	0.492	0.487
Firmicutes	0.037	0.095	0.023	0.173	0.164	0.133	0.156	0.102	0.074
Cyanobacteria	0.198	0.227	0.307	0.001	0.002	0.002	0.017	0.028	0.003
Planctomycetes	0.102	0.167	0.132	0.002	0.003	0.004	0.023	0.009	0.001
Bacteria_Unclassified	0.002	0.010	0.060	0.001	0.000	0.001	0.219	0.060	0.039
Bacteroidetes	0.007	0.030	0.014	0.082	0.103	0.079	0.007	0.001	0.003
Actinobacteria	0.009	0.013	0.013	0.009	0.011	0.005	0.005	0.003	0.000
Unclassified	0.004	0.003	0.004	0.002	0.005	0.044	0.004	0.001	0.001
Verrucomicrobia	0.008	0.017	0.000	0.000	0.000	0.000	0.000	0.000	0.000

Table. S3 Gut microbiome composition of the three cold-water fishes among different species and intestinal sections at family level (top 30).

Family level	*S.Wangchiachii*			*P. pingi*			*S.kozlovi*		
	**Foregut**	**Midgut**	**Hindgut**	**Foregut**	**Midgut**	**Hindgut**	**Foregut**	**Midgut**	**Hindgut**
Fusobacteriaceae	0.001	0.043	0.212	0.261	0.147	0.263	0.256	0.491	0.481
Aeromonadaceae	0.000	0.002	0.005	0.187	0.363	0.271	0.192	0.207	0.252
SubsectionIII_FamilyI	0.159	0.193	0.281	0.000	0.001	0.001	0.008	0.023	0.003
Planctomycetaceae	0.101	0.167	0.132	0.001	0.003	0.004	0.023	0.009	0.001
Bacteria_Unclassified	0.002	0.010	0.060	0.001	0.000	0.001	0.219	0.060	0.039
Enterobacteriaceae	0.001	0.004	0.002	0.166	0.049	0.018	0.039	0.056	0.015
Rhodobacteraceae	0.121	0.067	0.028	0.011	0.014	0.008	0.028	0.009	0.001
Shewanellaceae	0.000	0.000	0.000	0.040	0.003	0.108	0.010	0.012	0.108
Phyllobacteriaceae	0.189	0.040	0.020	0.000	0.001	0.001	0.002	0.000	0.000
Clostridiaceae 1	0.014	0.017	0.007	0.042	0.007	0.034	0.044	0.024	0.028
Erysipelotrichaceae	0.003	0.015	0.005	0.051	0.083	0.041	0.006	0.010	0.002
Carnobacteriaceae	0.003	0.003	0.001	0.001	0.002	0.000	0.097	0.065	0.042
Lactobacillaceae	0.004	0.033	0.004	0.053	0.032	0.027	0.004	0.000	0.001
Bacteroidales S24-7 group	0.001	0.011	0.004	0.050	0.056	0.031	0.003	0.000	0.001
Legionellaceae	0.026	0.115	0.009	0.000	0.000	0.000	0.001	0.000	0.000
Sphingomonadaceae	0.039	0.031	0.037	0.002	0.004	0.001	0.000	0.001	0.000
Bradyrhizobiaceae	0.085	0.008	0.004	0.000	0.000	0.000	0.000	0.000	0.000
Comamonadaceae	0.004	0.021	0.030	0.015	0.020	0.004	0.002	0.001	0.000
Unclassified	0.004	0.003	0.004	0.002	0.005	0.044	0.004	0.001	0.001
Rhizobiales Incertae Sedis	0.042	0.013	0.008	0.000	0.000	0.000	0.001	0.001	0.000
Acetobacteraceae	0.004	0.011	0.016	0.006	0.013	0.008	0.007	0.001	0.000
Prevotellaceae	0.000	0.001	0.001	0.012	0.021	0.020	0.002	0.000	0.001
Lachnospiraceae	0.001	0.013	0.001	0.012	0.010	0.010	0.002	0.001	0.001
Xanthomonadaceae	0.009	0.007	0.006	0.005	0.012	0.005	0.005	0.002	0.000
MNG7	0.048	0.000	0.000	0.000	0.000	0.000	0.001	0.000	0.000
SubsectionI_FamilyI	0.024	0.016	0.007	0.000	0.000	0.000	0.001	0.000	0.000
Neisseriaceae	0.000	0.010	0.004	0.006	0.001	0.005	0.005	0.004	0.011
Cyanobacteria_Unclassified	0.011	0.013	0.016	0.000	0.000	0.000	0.002	0.005	0.001
Bacteroidaceae	0.000	0.001	0.001	0.013	0.017	0.014	0.001	0.000	0.000
Alcaligenaceae	0.002	0.004	0.003	0.007	0.026	0.004	0.000	0.000	0.000

Table. S4 Gut microbiome composition of the three cold-water fishes among different species and intestinal sections at genus level (top 50).

Genus level	*S.Wangchiachii*			*P. pingi*			*S.kozlovi*		
	**Foregut**	**Midgut**	**Hindgut**	**Foregut**	**Midgut**	**Hindgut**	**Foregut**	**Midgut**	**Hindgut**
Cetobacterium	0.001	0.043	0.212	0.261	0.147	0.263	0.256	0.491	0.481
Aeromonas	0.000	0.002	0.005	0.187	0.363	0.271	0.192	0.207	0.252
SubsectionIII_FamilyI_Unclassified	0.150	0.184	0.269	0.000	0.001	0.000	0.008	0.023	0.003
Bacteria_Unclassified	0.002	0.010	0.060	0.001	0.000	0.001	0.219	0.060	0.039
Plesiomonas	0.001	0.003	0.001	0.163	0.042	0.017	0.037	0.056	0.014
Pirellula	0.065	0.113	0.091	0.000	0.000	0.000	0.010	0.004	0.001
Shewanella	0.000	0.000	0.000	0.040	0.003	0.108	0.010	0.012	0.108
Mesorhizobium	0.187	0.039	0.019	0.000	0.000	0.000	0.002	0.000	0.000
Carnobacterium	0.003	0.002	0.001	0.000	0.002	0.000	0.097	0.065	0.042
Clostridium sensu stricto 1	0.012	0.015	0.006	0.042	0.007	0.034	0.044	0.024	0.028
Rhodobacter	0.089	0.056	0.019	0.001	0.001	0.002	0.022	0.006	0.000
Lactobacillus	0.004	0.033	0.004	0.053	0.032	0.027	0.004	0.000	0.001
Bacteroidales S24-7 group_norank	0.001	0.011	0.004	0.050	0.056	0.031	0.003	0.000	0.001
Legionella	0.026	0.115	0.009	0.000	0.000	0.000	0.001	0.000	0.000
Erysipelotrichaceae_uncultured	0.001	0.008	0.002	0.031	0.025	0.021	0.005	0.010	0.002
Bosea	0.084	0.007	0.003	0.000	0.000	0.000	0.000	0.000	0.000
Polymorphobacter	0.031	0.022	0.032	0.000	0.000	0.000	0.000	0.001	0.000
Faecalibaculum	0.000	0.004	0.001	0.013	0.049	0.009	0.001	0.000	0.000
Unclassified	0.004	0.003	0.004	0.002	0.005	0.044	0.004	0.001	0.001
Phreatobacter	0.042	0.012	0.008	0.000	0.000	0.000	0.001	0.001	0.000
Planctomyces	0.011	0.020	0.018	0.000	0.000	0.001	0.006	0.002	0.000
MNG7_norank	0.048	0.000	0.000	0.000	0.000	0.000	0.001	0.000	0.000
Cyanobacteria_Unclassified	0.011	0.013	0.016	0.000	0.000	0.000	0.002	0.005	0.001
Bacteroides	0.000	0.001	0.001	0.013	0.017	0.014	0.001	0.000	0.000
Delftia	0.001	0.003	0.005	0.015	0.020	0.004	0.001	0.000	0.000
Deefgea	0.000	0.010	0.004	0.006	0.001	0.005	0.005	0.002	0.006
Achromobacter	0.000	0.001	0.002	0.007	0.026	0.002	0.000	0.000	0.000
Gleocapsa	0.021	0.012	0.004	0.000	0.000	0.000	0.000	0.000	0.000
Proteobacteria_Unclassified	0.000	0.006	0.025	0.000	0.000	0.000	0.000	0.003	0.001
Pelomonas	0.001	0.013	0.021	0.000	0.000	0.000	0.000	0.000	0.000
Tabrizicola	0.020	0.006	0.003	0.000	0.000	0.000	0.003	0.001	0.000
Acetobacter	0.001	0.001	0.000	0.005	0.012	0.007	0.006	0.000	0.000
Zavarzinella	0.013	0.011	0.008	0.000	0.000	0.000	0.000	0.000	0.000
Planctomycetaceae_uncultured	0.006	0.010	0.006	0.001	0.002	0.001	0.003	0.001	0.000
Meganema	0.014	0.005	0.003	0.000	0.000	0.000	0.005	0.001	0.000
Brevundimonas	0.001	0.001	0.002	0.004	0.018	0.002	0.000	0.000	0.000
Pannonibacter	0.000	0.000	0.000	0.010	0.012	0.003	0.000	0.000	0.000
Roseococcus	0.002	0.009	0.014	0.000	0.000	0.000	0.000	0.000	0.000
Pandoraea	0.001	0.001	0.001	0.005	0.015	0.002	0.000	0.000	0.000
Flavobacterium	0.003	0.011	0.006	0.000	0.000	0.000	0.000	0.000	0.001

Alloprevotella	0.000	0.001	0.000	0.008	0.007	0.005	0.000	0.000	0.000
Arenimonas	0.007	0.006	0.004	0.000	0.000	0.000	0.002	0.002	0.000
Gemmata	0.004	0.008	0.006	0.000	0.000	0.000	0.002	0.001	0.000
Rhodobacteraceae_Unclassified	0.010	0.004	0.004	0.000	0.000	0.000	0.002	0.001	0.000
Luteolibacter	0.006	0.013	0.000	0.000	0.000	0.000	0.000	0.000	0.000
Cyanobacteria_norank	0.004	0.004	0.002	0.000	0.000	0.001	0.006	0.000	0.000
Ralstonia	0.003	0.005	0.003	0.000	0.000	0.000	0.004	0.001	0.000
Allobaculum	0.000	0.000	0.000	0.002	0.005	0.009	0.000	0.000	0.000
Lachnospiraceae_uncultured	0.000	0.007	0.000	0.004	0.003	0.002	0.001	0.000	0.000
Leptolyngbya	0.007	0.007	0.003	0.000	0.000	0.000	0.000	0.000	0.000

Table. S5 Alpha diversity of the three cold-water fishes.

Diversity indices	***S.Wangchiachii***			***P. pingi***			***S.kozlovi***		
	**Foregut**	**Midgut**	**Hindgut**	**Foregut**	**Midgut**	**Hindgut**	**Foregut**	**Midgut**	**Hindgut**
Observed OTUs	954 ± 364	1173 ± 206	773 ± 452	343±154	377±216	397±342	327±309	221±197	143±133
Chao 1 index	1182±339	1395±211	995±485	411±160	446±237	459±365	457±346	343±254	224±237
Shannon	4±1	4±1	3±1	2±1	3±2	3±2	2±1	1±0.5	1±0.3
Phylogenetic diversity	66±22	77±12	53±28	41±15	44±18	39±24	32±20	23±12	19±11

Table. S6 Niche breadth of the three cold-water fishes.

Diversity indices	***S.Wangchiachii***			***S.kozlovi***			***P. pingi***		
	**Foregut**	**Midgut**	**Hindgut**	**Foregut**	**Midgut**	**Hindgut**	**Foregut**	**Midgut**	**Hindgut**
Niche breadth	12 ±7	12 ±8	6±3	3 ±1	3 ±1	3 ±1	7 ±7	12 ±13	11 ±14

QIIME analysis

Raw Data Quality Control

In order to obtain more accurate and reliable results in subsequent bioinformatics analysis, the raw data will be pre-processed to get clean data by three steps as following: 1) Raw reads filtration: Raw reads were firstly filtered by Trimmomatic v0.33. Then the primer sequences were identified and removed by cutadapt 1.9.1 and a custom Perl script, which finally generated high-quality reads without primer sequences. 2) High-quality reads assembly: Based on overlapping sequences, high-quality reads were assembled by FLASH v1.2.7, which generated clean reads. 3) Chimeric removal: Chimeric sequences were identified and removed by UCHIME v4.2, generating effective reads.

OTU clustering and species annotation

Operational Taxonomic Units (OTUs) refers to a cluster of sequences used to define a group (e.g species, genus, strain, etc) in phylogenetic studies or population genetic studies. Sequences with ≥97% similarity were assigned to the same OTUs using using USEARCH (vsesion 10 http://drive5.com/uparse/). A representative sequence for each OTU was annotated with threshold 0.8 using UCLUST v1.2.22q by searching the SILVA database. For comparisons between samples, the OTU abundances were normalized by the number obtained from the sample with the lowest counts.
